# Mortality Statistics, Chicago

**Published:** 1875-07

**Authors:** 


***Chicago Mortality Report for May, 1875.*** Reported by Dr. BEN. C. MILLER, Sanitary Superintendent.

| | |
|---|---|
| Accident, burns | 2 |
| " crushed | 2 |
| " chloroform | 1 |
| " drowning | 5 |
| " fall | 2 |
| " in planing mill | 1 |
| " kicked by horse | 1 |
| " street cars | 1 |
| " railroad | 2 |
| " scalding | 2 |
| " smothered | 1 |
| Abscesses | 2 |
| Anæmia | 1 |
| Abortion | 1 |
| Angina pectoris | 1 |
| Apoplexy | 9 |
| Asthma | 4 |
| Bowels, hæmorrhage of | 1 |
| " ulceration of | 1 |
| Brain, congestion of | 6 |
| " paralysis of | 2 |
| " hæmorrhage of | 2 |
| " inflammation of | 5 |
| Bronchitis | 13 |
| " capillary | 5 |
| Cancer | 2 |
| " of breast | 1 |
| " of liver | 3 |
| " of rectum | 1 |
| " of stomach | 3 |
| " of uterus | 3 |
| Child birth | 2 |
| Cholera infantum | 7 |
| Consumption | 72 |
| Convulsions | 77 |
| " puerperal | 1 |
| Croup | 11 |
| Cyanosis | 1 |
| Cystitis | 1 |
| Debility, general | 8 |
| Delirium tremens | 1 |
| Diabetes | 2 |
| Diarrhœa | 3 |
| Diphtheria | 7 |
| Dropsy, general | 8 |
| " ovarian | 1 |
| Dysentery | 2 |
| Embolism | 1 |
| Enteritis | 15 |
| Erysipelas | 1 |
| Exhaustion | 2 |
| Fever, intermittent | 1 |
| " puerperal | 7 |
| " remittent | 3 |
| " scarlet | 21 |
| " typhoid | 9 |

| | |
|---|---|
| Gastritis | 1 |
| Gravel | 1 |
| Hæmorrhage | 1 |
| Heart, disease of | 7 |
| " organic disease of | 1 |
| " hypertrophy of | 2 |
| " rheumatism of | 2 |
| " valvular disease of | 3 |
| Hepatitis | 3 |
| Hernia, strangulated | 1 |
| " incarcerated | 1 |
| Hydrocephalus | 8 |
| Inanition | 13 |
| Intemperance | 1 |
| Intussusception | 1 |
| Insanity | 1 |
| Kidneys, Bright's disease of | 5 |
| " disease of | 4 |
| " inflammation of | 1 |
| Laryngitis | 1 |
| Leucocythemia | 2 |
| Liver, cirrhosis of | 5 |
| Lungs, congestion of | 6 |
| " œdema of | 2 |
| " hæmorrhage of | 1 |
| " emphysema of | 1 |
| " paralysis of | 1 |
| Mania | 1 |
| Metro peritonitis | 3 |
| Measles | 26 |
| Morbus coxarius | 1 |
| Meningitis | 17 |
| " cerebro-spinal | 8 |
| " tubercular | 3 |
| Mumps | 1 |
| Metritis | 2 |
| Old age | 8 |
| Paralysis | 2 |
| Pericarditis | 1 |
| Peritonitis | 11 |
| " puerperal | 2 |
| Pneumonia | 45 |
| " typhoid | 7 |
| Pleurisy | 2 |
| Pyæmia | 1 |
| Rheumatism | 1 |
| Scrofula | 2 |
| Septicæmia | 1 |
| Small-Pox | 1 |
| Spine, caries of | 1 |
| " injury to | 1 |
| Stomatitis | 2 |
| Suicide, by shooting | 1 |
| " by hanging | 2 |
| " by drowning | 2 |
| " by coal gas | 1 |

| | | | |
|---|---|---|---|
| Syphilis | 1 | Vitality deficient | 1 |
| Tabes mesenterica | 8 | Whooping cough | 4 |
| Teething | 6 | | |
| Trismus | 1 | Total | 603 |

Premature births, 5; Still births, 61. Total, 66.

COMPARISON.

Deaths in May, 1875, 603; in April. 1875, 646. Decrease, 43. Deaths in May, 1874, 507. Increase, 96.

AGES.

| | | | |
|---|---|---|---|
| Under one year | 177 | Thirty years to forty | 53 |
| One year to two | 63 | Forty " " fifty | 40 |
| Two years to three | 30 | Fifty " " sixty | 31 |
| Three " " four | 20 | Sixty " " seventy | 27 |
| Four " " five | 10 | Seventy " " eighty | 19 |
| Five " " ten | 26 | Eighty " " ninety | 6 |
| Ten " " twenty | 26 | Ninety " " one hundred | 1 |
| Twenty " " thirty | 74 | Total | 603 |

| | | | | | |
|---|---|---|---|---|---|
| White | 597 | Males | 323 | Married | 193 |
| Colored | 6 | Females | 280 | Single | 410 |
| Total | 603 | Total | 603 | Total | 603 |

NATIVITIES.

| | | | | | |
|---|---|---|---|---|---|
| Bohemia | 12 | France | 1 | Poland | 2 |
| Canada | 5 | Germany | 74 | Scotland | 2 |
| Native—Chicago | 78 | Holland | 2 | Sweden | 6 |
| Foreign, " | 213 | Ireland | 53 | Switzerland | 2 |
| U. States, other parts | 114 | Italy | 1 | Unknown | 6 |
| Denmark | 4 | Norway | 12 | | |
| England | 15 | Orkney Islands | 1 | Total | 603 |

Deaths daily, 19½. Mean thermometer, 55°.4 Rain fall, 3.64 inches.

MORTALITY BY WARDS.

| Wards. | No. Deaths. | Pop. in 1874. | Percentage. | Wards. | No. Deaths. | Pop. in 1874. | Percentage. |
|---|---|---|---|---|---|---|---|
| 1 | 6 | 5,725 | one death in 954 | 11 | 16 | 14,022 | one death in 876 |
| 2 | 1 | 4,830 | " " 4,830 | 12 | 16 | 16,792 | " " 1,050 |
| 3 | 18 | 14,861 | " " 826 | 13 | 16 | 17,892 | " " 1,119 |
| 4 | 9 | 15,361 | " " 1,707 | 14 | 17 | 16,720 | " " 984 |
| 5 | 25 | 20,078 | " " 803 | 15 | 95 | 45,545 | " " 480 |
| 6 | 53 | 35,916 | " " 678 | 16 | 31 | 21,922 | " " 707 |
| 7 | 55 | 31,722 | " " 577 | 17 | 32 | 20,777 | " " 649 |
| 8 | 38 | 29,143 | " " 767 | 18 | 31 | 21,392 | " " 690 |
| 9 | 42 | 31,654 | " " 754 | 19 | 4 | 4,677 | " " 1,169 |
| 10 | 14 | 17,385 | " " 1,242 | 20 | 7 | 8,995 | " " 1,285 |

Ratio of deaths to population in 1874, one death in 655⅘.

| | | | |
|---|---|---|---|
| No. deaths in Wards | 526 | Hospital for Women and Children | 1 |
| Accidents | 20 | Protestant Orphan Asylum | 1 |
| Bridewell | 1 | St. Joseph's Hospital | 3 |
| County Hospital | 12 | St. Luke's Hospital | 3 |
| Foundlings' Home | 17 | Suicides | 6 |
| Hospital Alexian Bros. | 6 | | |
| Mercy Hospital | 7 | Total | 603 |

